# Calculation of Evolutionary Correlation between Individual Genes and Full-Length Genome: A Method Useful for Choosing Phylogenetic Markers for Molecular Epidemiology

**DOI:** 10.1371/journal.pone.0081106

**Published:** 2013-12-03

**Authors:** Shuai Wang, Xuenong Luo, Wei Wei, Yadong Zheng, Yongxi Dou, Xuepeng Cai

**Affiliations:** State Key Laboratory of Veterinary Etiological Biology, Lanzhou Veterinary Research Institute, Chinese Academy of Agricultural Sciences, Lanzhou, Gansu, China; George Washington University, United States of America

## Abstract

Individual genes or regions are still commonly used to estimate the phylogenetic relationships among viral isolates. The genomic regions that can faithfully provide assessments consistent with those predicted with full-length genome sequences would be preferable to serve as good candidates of the phylogenetic markers for molecular epidemiological studies of many viruses. Here we employed a statistical method to evaluate the evolutionary relationships between individual viral genes and full-length genomes without tree construction as a way to determine which gene can match the genome well in phylogenetic analyses. This method was performed by calculation of linear correlations between the genetic distance matrices of aligned individual gene sequences and aligned genome sequences. We applied this method to the phylogenetic analyses of porcine circovirus 2 (PCV2), measles virus (MV), hepatitis E virus (HEV) and Japanese encephalitis virus (JEV). Phylogenetic trees were constructed for comparisons and the possible factors affecting the method accuracy were also discussed in the calculations. The results revealed that this method could produce results consistent with those of previous studies about the proper consensus sequences that could be successfully used as phylogenetic markers. And our results also suggested that these evolutionary correlations could provide useful information for identifying genes that could be used effectively to infer the genetic relationships.

## Introduction

**Figure 1:**
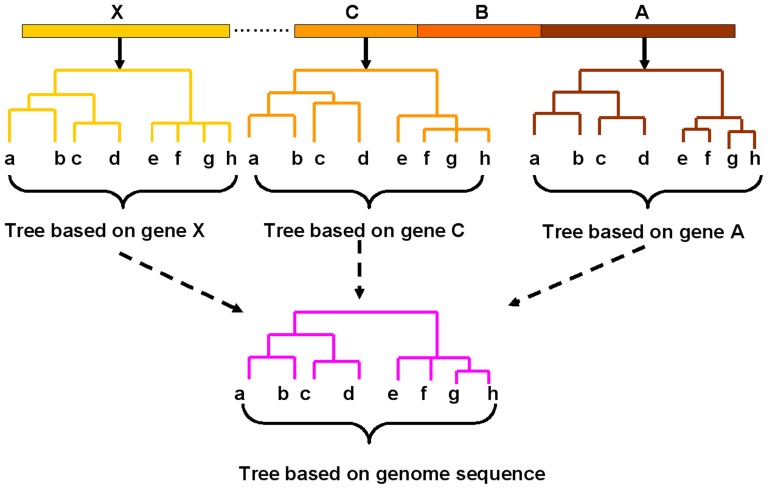
Discordance among trees based on different sequences used to estimate the evolutionary relationships of viral strains. The letters (A, B, C... X) represent genes (or genomic regions) from a hypothetical viral genome. The trees constructed from the different gene sequences show discordant topologies and tree branch lengths with each other. And the discordance can involve both the topology and lengths of different branches.

The phylogenetic analysis of viruses is a useful way to estimate their evolutionary relationships and assign genotypes. Until recently, the method commonly used for molecular phylogenetic studies for many viruses typically involved sequencing a gene or partial genomic region (phylogenetic marker) in individual representatives of a collection of strains and inferring a “gene tree” based on these sequences and declaring the gene tree to be the estimate of the tree of strain relationships [1]–[4]. However, many studies have shown that the evolutionary histories of some individual genes or genomic regions may not be identical to each other within many viruses, which may be due to the recombination, reassortment and selection pressure [5]–[15]. Therefore, several methods such as phylogenetic networks have been developed to infer these evolutionary processes including the recombination events. However, all these analyses could only provide accurate estimates only when all the whole-genome sequences are available [13]. In a molecular epidemiological survey, it is still expensive work to sequence the genome of every strain involved especially when the viral genomes are large, despite the decreasing cost of sequencing. For many viruses, a proper marker region used for the phylogenetic analysis can be still preferable in practical applications to infer the genetic relationships despite the fact that it may sometimes give some misleading assessments (Figure 1).

Given that full-genome sequences can provide the relatively more reliable information about genetic relationships between different virus isolates for many viruses, a region that can reconstruct a tree similar to the genome tree would be a preferred candidate for the phylogenetic marker [6], [7], [13], [16]–[21]. For many viruses, some viral genomic regions have been shown to act as good phylogenetic markers because the topologies of the genome tree and the trees constructed from those individual genes or genomic regions are similar [6], [7], [9], [15], [16], [22].

In this study, we used a statistical method without tree construction,based on the genetic distances between nucleotide sequences, as an alternative way to estimate which genomic region can match the whole genome well in a phylogenetic analysis. This method functioned like the “mirrortree” method for predicting protein-protein interactions which was successful to measure quantitatively the correlation between the phylogenetic trees of two proteins or domains [23]–[26].

The correlations of evolutionary rates between the full-length viral genome and individual genes sequences were calculated as linear correlation coefficients between the genetic distance matrices. The genomes of porcine circovirus 2 (PCV2), measles virus (MV), hepatitis E virus (HEV) and Japanese encephalitis virus (JEV) were used in our calculations. All of these viruses have been studied in detail to select the appropriate phylogenetic markers by comparing the trees constructed with individual genomic regions and with whole-genomes [6], [7], [9], [16], [27]. Our results suggested that the correlation coefficients were helpful in estimating the evolutionary relationships between full-length genome and their genomic regions and could have the potential as an alternative way of providing useful information for selecting the candidate for phylogenetic markers for molecular epidemiology.

## Materials and Methods

### 2.1 Data collection

The viral genomes of PCV2, MV, HEV and JEV were obtained from GenBank (http://www.ncbi.nlm.nih.gov), partly according to the previously published studies. All these sequences would be compared with the reference genomes curated in the GenBank and the sequences containing gaps in genomic regions would be discarded unless they were clearly confirmed by other studies. And the sequences that have been fully reported in previous studies, with clear backgrounds and well annotated genome structures were preferred. For the viral strains with conflicting sequences, the sequence meeting the above criteria described above was selected. If the whole-genome sequences of several strains were identical, only one whole-genome sequence was retained. All data were collected before 1 October, 2013. Firstly, 43 PCV2 strains, 28 MV strains, 33 HEV strains and 30 JEV strains were used in the general calculations (Table S1 and Table S2). To allow comparisons to be made, large sets of PCV2 sequences (123 strains and 224 strains; Text S1) were also obtained for another calculation. The genomic region sequences for each virus were obtained depending on reference genomes annotated in the NCBI. The main genes (rep and cap) of PCV2, genes coding for structural proteins (N, P, M, F, H and L) and non-structural proteins (C and V) of MV, several genomic regions (MJ-C, GO, KLY-B, MXJ and SGG-A) depending on the literature [7] from HEV and genes coding for structural proteins (E, PreM and cap) and non-structural protein (NS1, NS2a, NS2b, NS3, NS4a, NS4b and NS5) of JEV were obtained from the selected genomes respectively. The sizes of these individual genes or genomic regions were given in Table S3.

### 2.2 Multiple sequence alignments and phylogenetic analysis of individual genes and full genome

The nucleotide sequences of all the genomes and individual genes of each virus were aligned separately using ClustalW [28], followed by manual adjustment using known landmarks in the viral genomes. The Findmodel (http://www.hiv.lanl.gov/content/sequence/findmodel/findmodel.html) was employed to estimate a proper evolutionary model to construct phylogenetic trees. The phylogenetic trees were constructed by Neighbor-joining method based on the Tamura 3-parameter substitution model implemented in the MEGA (version 5) [29] with 1000 bootstrap replicates. The genome and gene nucleotide sequences of the 43 PCV2 strains, 28 MV strains, 33 HEV strains and 30 JEV strains were all involved in the alignments and tree constructions. Multiple sequence alignments for two large sets of PCV2 sequences (123 strains and 224 strains) were also performed.

### 2.3 Statistical method used to calculate evolutionary correlations

**Figure 2:**
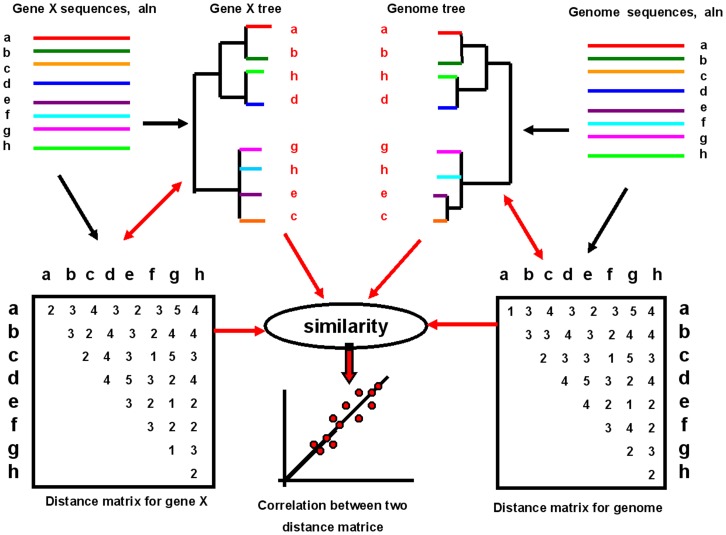
Methodology for estimating the evolutionary relationships between individual genes and their genome. Multiple sequence alignments were performed based on the nucleotide sequences of gene X and its genome nucleotide sequences. The distance matrices were constructed from the alignments. Finally, the similarity between the two distance matrices was evaluated with a linear correlation coefficient. Note that no construction of phylogenetic trees was necessary in the calculation.

In order to estimate the evolutionary relationship between two nucleotide sequences, we introduced the r-value (Pearson correlation coefficient) [30] for calculation using pairwise distance matrices. As the basic data for certain algorithms to construct the trees, the pairwise distances determine the tree topology [31]. Therefore, the correlation coefficient between two distance matrices can be used to estimate the similarity of two trees (Figure 2) [23], [24], [32]. Here, we firstly used this statistical method to estimate the evolutionary correlation levels between genome sequences and individual gene sequences so as to choose the proper consensus region for inferring the exact genetic relationship between viral isolates. The calculation was performed as described below:

Evolutionary pairwise distance matrices were constructed with MEGA (version 5) based on the former multiple sequence alignments, using the Tamura 3-parameter model method [33]. For gene X from its whole genome Y, the evolutionary linear correlation coefficient r (Pearson’s correlation coefficient) between them was calculated according to the following equation [30]:
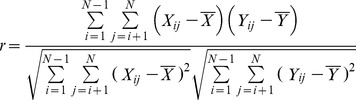



where X_ij_ represents the distance from i virus strain to j virus strain in distance matrix of gene X; 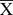 represents the mean of all X_ij_ values; Y_ij_ represents the distance of strain i to strain j in the distance matrix of genome Y; 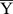
represents the mean of all Y_ij_ values; and N represents the number of viral strains in the matrices.

The significance of the computed r value was assessed by estimating the probability of obtaining the observed value of r by chance (p-value). The correlation coefficient r-values between genes and the complete genome of each virus were respectively calculated based on all the sets of strains collected.

### 2.4 Analysis based on different statistical samples

As a statistical parameter, the r-value may be affected by the samples sizes and the sample sources. In order to evaluate the effect of samples size on the r-values, calculations based on different numbers of strains which was randomly selected were performed in PCV2 (sample size: 10, 15, 20, 25, 30, 35 and 40), MV (sample size: 10, 15, 20, 25 and 28), HEV (sample size: 10, 15, 20, 25 and 30) and JEV (sample size: 10, 15, 20, 25 and 30). And random samples (sample size: 5, 10, 15, 20, and 30) from the obtained PCV2 strains were separately selected from the obtained PCV2 strains five times for each sample size to assess the effects of sample source on the r values.

## Results

### 3.1 Discordance between full-length genome tree and individual gene trees

**Figure 3:**
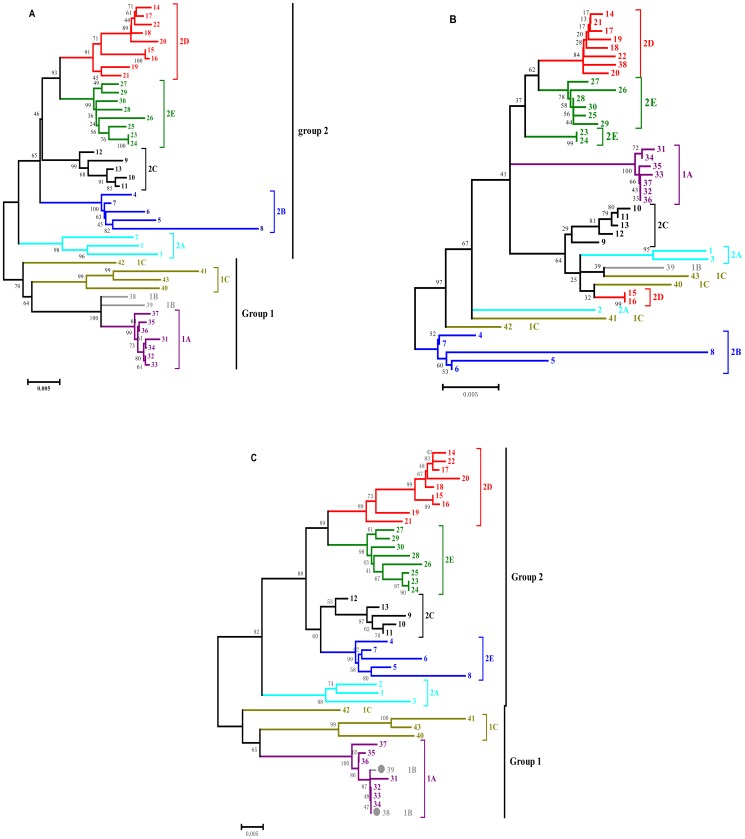
Phylogenetic analysis of PCV2 strains based on the genome sequence, rep gene sequence and cap gene sequence. The A, B, and C represent trees constructed from the genome sequence, rep gene sequence and cap gene sequence respectively. The evolutionary relationships among Circovirus groups inferred from cap gene sequence could match that from genome sequence very well. Great discordance exists between the gene rep sequence and genome sequence.

The phylogenetic trees based on the genomes and each gene of the four viruses were constructed separately for comparison (Figure S1, Figure S2, Figure S3, and Figure 3). For all the four viruses (Table S1 and Table S2), the individual gene trees differed not only from the corresponding genome tree, but from the trees constructed from the other genes in the same genome, in both their topologies and branch lengths in a way.

For PCV2, the cap gene shared a more similar tree with the genome than the rep gene both in topology and branch length obviously (Figure 3). Few discrepancies in the definition of groups and clusters were observed between the phylogenetic trees of the cap gene and the whole PCV2 genome except in the genotype 1B cluster (strain NO. 38 and 39) for which the tree based on cap gene failed to differentiate its members precisely (Figure 3). However, the tree based on the rep gene presented much incongruence with the cap and the complete genome trees, leading to drastically disordered groupings for viral strains.

The MV trees based on the P gene, M gene, V gene and C gene could not match the genome trees very well. However, the L gene, N gene, H gene and F gene trees could reproduce the topology of the full-length genome tree more similar than others with reliable bootstrap support values (Figure S1).

In HEV, the trees of region GO and KLY-B differed dramatically from the genome trees in topologies, which resulted in misleading inferences on genetic relationships of some strains. However, it was hard to estimate which of the regions SGG-A, MJ-C and MXJ produced a tree that was most similar to the full-genome tree (Figure S3).

In JEV, the trees of gene E, NS1 and NS5 could agree well with the genome tree. However, it was hard to access which one from the genes NS2a, NS2b, NS3, NS4b and PreM could yield a tree more concordant with the genome tree due to different discordance involving different virus strains. The cap gene tree displayed topology obviously disagreeing with the genome tree and other gene trees (Figure S2).

### 3.2 Evolutionary correlation between genome and individual genes

The evolutionary correlation coefficients between the genome and individual genes (or genomic regions) of each virus varied (Table 1, Table S4, Table S5, Table S6 and Table S7). For PCV2, the cap gene shared a much higher r-value with the genome than did the rep gene, for both the small sample (43 strains) and the large samples (123 and 224 strains) of sequences.

**Table 1:** Evolutionary correlations between the genome sequence and individual gene sequences based on whole sets of viral strains.

Genes of JEV	cap	E	NS1	NS2a	NS2b	NS3	NS4a	NS4b	NS5	PreM
R-values (30)	0.927	0.996	0.993	0.987	0.987	0.986	0.979	0.992	0.996	0.987
Genes of MV	F	H	L	M	N	P	V	C		
R-values (28)	0.973	0.979	0.992	0.957	0.981	0.953	0.929	0.877		
Genes of HEV	MJ-C	GO	KLY-B	MXJ	SGG-A					
R-values (33)	0.983	0.938	0.933	0.983	0.972					
Genes for PCV2	cap	rep								
R-values (43)	0.940	0.766								
R-values (120)	0.940	0.767								
R-values (220)	0.961	0.790								

The numbers in the parentheses indicate the numbers of viral strains used for the calculations.

For the trees based on sequences from MV, the topologies did not differ dramatically among them. However our data showed that the 8 genes shared different correlation coefficient values with the genome (Table 1). The L gene showed the highest r-value (0.992) with the full genome which was slightly higher than those of the N gene (0.981), H gene (0.979) and F gene (0.973). In addition, the r-values showed that the evolutionary rates of M, P, C and V gene related more distantly with the full genome.

Within the calculation for JEV (Table 1), the E gene and NS5 gene had the highest evolutionary correlation value (0.996) with the genome, slightly higher than that of NS1 (0.994) and that of NS4b (0.992). The r-values of NS2a, NS2b and PreM with the genome were all the same (0.987), while they were a little higher than the r-value of NS3 with the genome (0.986). The cap gene showed the lowest correlation values with the genome (0.927).

For HEV, all the correlation values were less than 0.990, relatively lower than those for the other viruses (Table 1). The MJ-C region had the highest value (0.983) with the genome whereas the KLY-B region had the lowest r-value (0.933).

The pairwise correlation values for genes in the same genome were also calculated (data not shown), and indicated that different genes or genomic regions from all the four viruses could underline inconsistent evolution with each other in some way. The evolutionary histories of genes could differ greatly. For instance, the cap and rep genes in PCV2 shared an evolutionary r-value of 0.55.

### 3.3 Analyses based on different statistical samples

**Figure 4:**
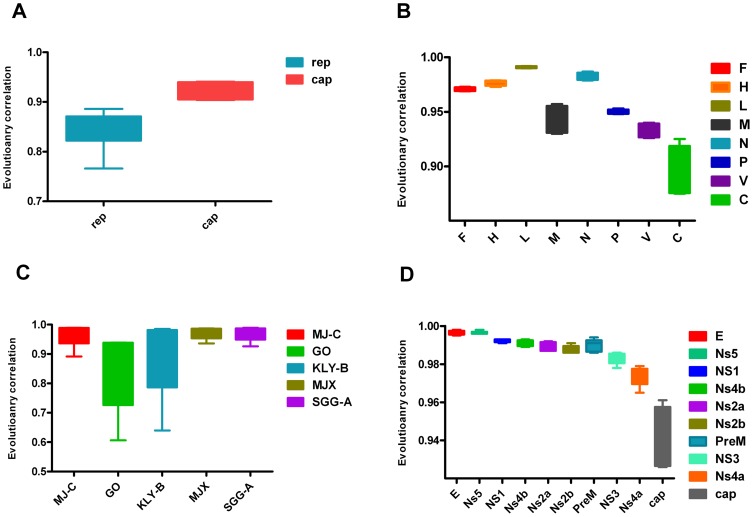
The r-values between different individual genes (or genomic regions) and genomes of the four viruses based on different sample sizes. The A, B, C and D represent r-values of PCV2, MV, HEV and JEV respectively in order. Each set of r-values between one individual genomic region and its genome for different number of viral strains was shown as boxes-and-whiskers plot. The whiskers go from the highest to lowest point.

**Figure 5:**
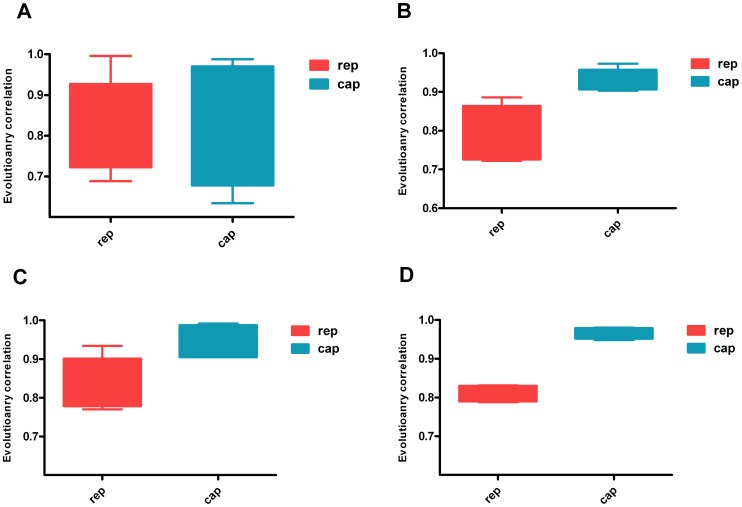
The r-values between individual genes and its whole genome of PCV2 based on different sample sources and sizes. The A, B, C and D represent r-values based on the genome samples of 5, 10, 15 and 20 strains respectively. Each set of data points (five points each set) comprises the r-values calculated from randomly selected samples of one specific sample number, presented as box-and-whisker plots. The whiskers go from the highest to the lowest point.

For each virus, the calculations for different numbers of viral genome samples and a random selection of PCV2 genome samples with the same size were carried out respectively. The r-values are shown as boxes-and- whiskers plots. Our results suggested that the r-values could vary with the sample selection to some degree. However, the trend for each gene was clear enough to estimate its reliability. The r-values of some genes were high and stable (higher and shorter appearance of boxes and whiskers), whereas the r-values of other genes were more changeable and lower (lower and longer appearance of boxes and whiskers) (Figure 4). As can be seen in Figure 5, when the r-values were calculated with five PCV2 strains, it was hard to determine which gene had higher correlation with genome because the r-values were highly variable across the different samples. However, when the sample number was greater than 10, the comparison became available with a clear trend in the r-values of the two genes. This was consistent with the results based on samples of different sizes for (Table S4, S5, S6 and S7) the four viruses, in which the trend was significant and similar when the sample size was greater than 10.

## Discussion

A phylogenetic analysis is commonly performed by inferring genetic relationships between viral strains from a tree based on one individual gene or genomic region [1]. However, the use of different genomic regions by different laboratories makes it difficult to compare their results and can even give misleading inferences. Unlike the previous tree comparison strategies, we tried to take advantage of a statistical method as an alternative way to provide information for selecting the phylogenetic markers.

This method functions like the “mirrortree” method, in which the similarity between trees is evaluated with a linear correlation coefficient using genetic distance matrices [23], [25]. The r-values from our calculations represented the similarities and agreed with the result from the direct comparisons of the trees (Figures 3, S1, S2, and S3). A higher r-value between the whole genome and one individual gene means a more correlated evolutionary rate between them. This method does not require the construction of phylogenetic trees and only the underlying distance matrices are analyzed, which makes this approach independent of any given distance-based tree-construction methods.

The L, F and H genes of MV showed the higher and more stable r-values with the genome, implying more related evolutionary rates with their genome sequence (Table 1 and Table S4). This is consistent with the fact that the N gene and H gene sequences are most commonly recommended for the genetic characterization of wild-type viruses [27], [34]-[36]. Although the L gene could reproduce the genome tree well, the large size of sequence limits its utility. For PCV2 which has the smallest genome in the *circoviridae* family, encoding only two major proteins (rep and cap), the cap gene correlated more strongly and more stably with the full genome than the rep gene in its evolutionary rate, which is consistent with our phylogenetic analysis based on tree comparison (Figure 3). This is fully supported by the study of Olvera et al [6], who considered that the cap gene was a more reliable phylogenetic marker for PCV2 strains because it was able to reconstruct the same tree as the whole viral genome. The cap gene shows much greater variability than the rep gene, which may be due to the fact that the cap protein is the most exposed part of the virus and is the most likely to interact with the host immune system [6], [37]. In JEV, the E gene has been given precedence when there is discordance between the E and other genes because it has been shown to be a better predictor of phylogenies determined by complete genomes [9]. And the NS1 gene and NS5 gene have been also proved as good reference genes for genotypic assignment [16], [38], [39]. In this study, we found that the E and NS5 gene showed the strongest and relatively constant correlation values (0.996) with the full-length genome and the NS1 gene also show a higher r-value (0.993) with the genome (Figure 4). It has been known that some genomic regions for HEV genotyping can correlate well with the results from the phylogenetic analyses based on the complete genome [7], [40], [41]. The MJ-C region in the viral RdRp domain has been identified as a substitute for the full-length genome when assigning genotypes [7]. We have drawn the same conclusion as that study by calculating the evolutionary correlations between the HEV genome and the selected regions from the previous study. The MJ-C region showed the highest r-value (0.983) with the full genome, indicating the MJ-C region is evolving in a rate more correlatively with the HEV genome across all the selected regions. However, according to our results (Figure 4), all the regions of HEV selected for our analysis, including the MJ-C region, showed unstable evolutionary correlations with the genome based on different genome samples. This may suggest that they may be inappropriate for phylogenetic analysis, which was supported by the unreliable bootstrap values in the phylogenetic trees (Figure S3) and may arise partly from the limited informative sites in all the selected regions with sequences short in length.

A limited number of samples can negatively affect the sensitivity of the method. Our analysis of differently sized samples of viral strains, as well as random and repetitive sampling of PCV2, showed empirically that an insufficient sample size, which was less than 10, may negatively affect the stability and reliability of r-values (Figure 5 and Table S5). This may result from insufficient informative variation of each sequence for detection, the limitations of statistical method itself and/or the potential specific variability in evolutionary history. Consequently, an empirical restriction will be recommended that the minimum sample size is 10. Furthermore, no significant difference was found between the results based on a small number (43 strains) and those based on large numbers (123 and 224 strains) of PCV2 viruses. Another factor, the sample source also seemed to play an important role in the calculation for the r-values. While the r-values changed with the different selection of PCV2 strains, it had no influence on the comparison for judgment because of the clear trend between r-values when the size of sample was sufficiently large. Sufficient information can be provided by more representative strains from different lineages. Moreover, the comparison between the results from calculations with all the selected strains (Table 1) and those of several randomly selected groups with different numbers revealed that estimates from different sample sizes and sample sources (Figure 4 and Figure 5) were consistent with that from one specific calculation with enough number of representative strains. Therefore, a reliable estimate can be made when the calculation is based on a sufficient number of representative strains. In practice, the r-values would be calculated based on several groups of randomly selected strain sources with the sufficient number, which would yield a more reliable assessment.

Although a high consistency between some individual genes and their whole genome in referring phylogenetic relationships can be considered as a good signature of a phylogenetic marker for some viruses, more factors should be taken into consideration when determining the best phylogenetic markers, such as the phenotype, recombination, antigenicity and virulence [6], [13], [16]. Our method can only give estimates of the correlated evolution level between individual genomic regions and the whole genome of some viruses and then provide the useful information for determining the proper phylogenetic markers for molecular epidemiology. More comprehensive analyses must be performed to choose one specific genome region as a phylogenetic marker.

## Supporting Information

Figure S1 **Phylogenetic analyses of MV isolates based on the full-length genome and different individual genes.** The trees based on the P, M, V and C genes could not match the genome trees very well and presented discordant clusters for some strains. The L, N, H and F genes could reproduce the topology obtained with the full-length genome sequence more similar than others, and with reliable bootstrap support values.
(TIF)

Figure S2 **Phylogenetic analyses of JEV isolates based on the full-length genome and different individual genes**. The trees constructed from the E, NS1 and NS5 genes could agree well with the genome tree. It was hard to access which one from the genes NS2a, NS2b, NS3, NS4b and PreM could yield a tree most concordant with the genome tree due to different discordance involving different virus strains. The topology of the cap gene tree was clearly inconsistent with that of the genome tree and other gene trees.
(TIF)

Figure S3 **Phylogenetic analyses of HEV isolates based on the full-length genome and different individual genes**. The trees constructed from the regions GO and KLY-B differed dramatically from the genome trees in topology, which results in misleading infer on genetic relationships of some strains. It was difficult to determine which of the regions SGG-A, MJ-C, and MXJ generates the tree most similar to the genome tree.
(TIF)

Table S1 **Information about the MV, HEV and JEV isolates used in this study.**
(DOC)

Table S2 **Information about the PCV2 isolates used in this study.**
(DOC)

Table S3 **Sizes of the individual genes or genomic regions used in this study.**
(DOC)

Table S4 **Evolutionary correlation r values between the genome and individual genes of MV based on differently sized samples.**
(DOC)

Table S5 **Evolutionary correlation r values between the genome and individual genes of PCV2 based on differently sized samples.**
(DOC)

Table S6 **Evolutionary correlation r values between the genome and individual genes of JEV based on differently sized samples.**
(DOC)

Table S7 **Evolutionary correlation r values between the genome and individual genes of HEV based on differently sized samples.**
(DOC)

Text S1 **Accession numbers for the PCV2 strains used for the calculations based on large numbers of sequences.**
(DOC)
