# Supplementary material for: Calculation of Evolutionary Correlation between Individual Genes and Full-Length Genome: A Method Useful for Choosing Phylogenetic Markers for Molecular Epidemiology
Source: PLoS One. 2013 Dec 3;8(12):e81106. doi: 10.1371/journal.pone.0081106 (PMC3849185; doi:10.1371/journal.pone.0081106)
Supplement: Table S1 — Information about the MV, HEV and JEV isolates used in this study. (DOC) [file pone.0081106.s004.doc]

**Table S1 Information about the MV, HEV and JEV isolates used in this study.**

| Virus strains | Accession Number | Genotype | Virus strains | Accession Number | Genotype | |
| --- | --- | --- | --- | --- | --- | --- |
| MV strains used in this study | | | | | | |
| MVi/California.USA/16.03 | JN635410.1 | D7 | Shanghai-191 | [EU435017](http://www.ebi.ac.uk/ebisearch/redirect.ebi?url=http%3A%2F%2Fwww.ebi.ac.uk%2Fena%2Fdata%2Fview%2FEU435017&id=EU435017&requestFrom=field&rank=1&domain=emblrelease_standard&digest=8504406F7835DEE2790AA6F295A2476660B50118) | | A |
| MVi/New Jersey.USA/45.05 | [JN635408](http://www.ebi.ac.uk/cgi-bin/sva/sva.pl?search=Go&query=JN635408) | B3 | CAM-70 | [DQ345721](http://www.ebi.ac.uk/cgi-bin/sva/sva.pl?search=Go&query=DQ345721) | | A |
| MVi/Arizona.USA/11.08/2 | JN635406.1 | D5 | Changchun-47 | [EF033071](http://www.ebi.ac.uk/ebisearch/redirect.ebi?url=http%3A%2F%2Fwww.ebi.ac.uk%2Fena%2Fdata%2Fview%2FEF033071&id=EF033071&requestFrom=field&rank=1&domain=emblrelease_standard&digest=BF13D327AF8C97D586C5ECF2C634F4AE13594E1D) | | A |
| MVi/Virginia.USA/15.09 | [JN635404](http://www.ebi.ac.uk/ebisearch/redirect.ebi?url=http%3A%2F%2Fwww.ebi.ac.uk%2Fena%2Fdata%2Fview%2FJN635404&id=JN635404&requestFrom=field&rank=1&domain=emblrelease_standard&digest=8AA530D96CD124F5CB84FEBC1EFBCAA0200DFFFF) | D8 | Leningrad-4 | [AY730614](http://www.ebi.ac.uk/ebisearch/redirect.ebi?url=http%3A%2F%2Fwww.ebi.ac.uk%2Fena%2Fdata%2Fview%2FAY730614&id=AY730614&requestFrom=field&rank=1&domain=emblrelease_standard&digest=1479F0DB89329E1A76D141A17D7FB2B69926BCAB) | | A |
| MVi/New York.USA/26.09/3 | [JN635402](http://www.ebi.ac.uk/cgi-bin/sva/sva.pl?search=Go&query=JN635402) | D4 | T11wild | [AB481087](http://www.ebi.ac.uk/ebisearch/redirect.ebi?url=http%3A%2F%2Fwww.ebi.ac.uk%2Fena%2Fdata%2Fview%2FAB481087&id=AB481087&requestFrom=field&rank=1&domain=emblrelease_standard&digest=335EAC58E14884341AB339F9A88E11F4C120CEF5) | | D3 |
| MVi/Pennsylvania.USA/20.09 | [JN635411](http://www.ebi.ac.uk/cgi-bin/sva/sva.pl?search=Go&query=JN635411) | H1 | Schwarz | [AF266291](http://www.ebi.ac.uk/ebisearch/redirect.ebi?url=http%3A%2F%2Fwww.ebi.ac.uk%2Fena%2Fdata%2Fview%2FAF266291&id=AF266291&requestFrom=field&rank=1&domain=emblrelease_standard&digest=EE35A86CDCB7C0B18FF9231A73C95A37A80BB991) | | A |
| MVi/Texas.USA/4.07 | JN635407.1 | D8 | MVi/Zhejiang.CHN/7.05/4 | [DQ211902](http://www.ebi.ac.uk/ebisearch/redirect.ebi?url=http%3A%2F%2Fwww.ebi.ac.uk%2Fena%2Fdata%2Fview%2FDQ211902&id=DQ211902&requestFrom=field&rank=1&domain=emblrelease_standard&digest=65699B425C1F8186957B970DEE72FCB41EA94783) | | H1 |
| MVi/Washington.USA/18.08/1 | [JN635405](http://www.ebi.ac.uk/cgi-bin/sva/sva.pl?search=Go&query=JN635405) | D5 | i97-45881 | [DQ227319](http://www.ebi.ac.uk/ebisearch/redirect.ebi?url=http%3A%2F%2Fwww.ebi.ac.uk%2Fena%2Fdata%2Fview%2FDQ227319&id=DQ227319&requestFrom=field&rank=1&domain=emblrelease_standard&digest=6F7B488C687F70C51DA0FF5191C9ADA620DB66CD) | | D6 |
| MVi/Florida.USA/19.09 | [JN635403](http://www.ebi.ac.uk/cgi-bin/sva/sva.pl?search=Go&query=JN635403) | D4 | Davis87 | [EU293550](http://www.ebi.ac.uk/ebisearch/redirect.ebi?url=http%3A%2F%2Fwww.ebi.ac.uk%2Fena%2Fdata%2Fview%2FEU293550&id=EU293550&requestFrom=field&rank=1&domain=emblrelease_standard&digest=E2F89F78E90512BC9738823FB55CE58013B8A8AE) | | D3 |
| KS | HM439386.1 | B3 | WA.USA/17.98 | [DQ227321](http://www.ebi.ac.uk/ebisearch/redirect.ebi?url=http%3A%2F%2Fwww.ebi.ac.uk%2Fena%2Fdata%2Fview%2FDQ227321&id=DQ227321&requestFrom=field&rank=1&domain=emblrelease_standard&digest=2669B87BE36763317CF640B0807D745F1E005BD9) | | D6 |
| IMB-1 | [FJ161211](http://www.ebi.ac.uk/cgi-bin/sva/sva.pl?search=Go&query=FJ161211) | H1 | Ichinose-B95a | AB016162 | | D3 |
| Leningrad-16 | [JF727649](http://www.ebi.ac.uk/cgi-bin/sva/sva.pl?search=Go&query=JF727649) | A | Edmonston-Zagreb | [AY486084](http://www.ebi.ac.uk/ebisearch/redirect.ebi?url=http%3A%2F%2Fwww.ebi.ac.uk%2Fena%2Fdata%2Fview%2FAY486084&id=AY486084&requestFrom=field&rank=3&domain=emblrelease_standard&digest=4D12C5FA27BE68242A2AD67E32C20B5B0EBBE9C6) | | A |
| Schwarz FF-8 | AB591381.1 | A | Edmonston | AF266288.2 | | A |
| MVi/California.USA/8.04 | [JN635409](http://www.ebi.ac.uk/ebisearch/redirect.ebi?url=http%3A%2F%2Fwww.ebi.ac.uk%2Fena%2Fdata%2Fview%2FJN635409&id=JN635409&requestFrom=field&rank=1&domain=emblrelease_standard&digest=EBE7FCD8B07DB10F98F4D7858163F303E4BE9825) | D3 |  |  | |  |
| JEV Strains used in this study | | | | | | |
| Beijing-1 | L48961 | III | TL | AF098737 | | III |
| CH2195LA | AF221499 | III | BL06-54 | JF706271 | | I |
| FU | AF217620 | II | YN79Bao83 | JN381851 | | I |
| GP78 | AF075723 | III | LN0716 | JN381849 | | I |
| Ishikawa | AB051292 | I | XJP613 | EU693899 | | I |
| JaGAr01 | AF069076 | III | GX0519 | JN381835 | | I |
| JaOArS982 | NC_001437 | III | GZ56 | HM366552 | | I |
| K94P05 | AF045551 | I | HN0626 | JN381837 | | I |
| JKT6468 | AY184212 | IV | GS07TS11 | JN381843 | | I |
| P3 | U47032 | III | M28 | JF706279 | | I |
| SA14 | U14163 | III | ZMT | JF706283 | | I |
| TC | AF098736 | III | LFM | JN381863 | | I |
| P20778 | AF080251 | III | Fj02-29 | JF706273 | | I |
| JaOArS892 | M18370 | III | JH0418 | JN381855 | | I |
| JE RP-9 | AF014161 | III | SH0410 | JN381856 | | I |
| HEV strains used in this study | | | | | | |
| Bur82 | M73218 | 1a | SwineHEV | AF082843 | | 3a |
| Bur86 | D10330 | 1a | JKN-Sap | AB074918 | | 3a |
| Yam67 | AF459438 | 1a | JMY-Haw | AB074920 | | 3a |
| Hyderabad | AF076239 | 1a | HE-JA10 | AB089824 | | 3a |
| TK15-92 | AF051830 | 1a | JRA1 | AP003430 | | 3b |
| Madras | X99441 | 1a | JJT-Kan | AB091394 | | 3b |
| pSK-HEV-2 | AF444002 | 1a | swJ570 | AB073912 | | 3c |
| KS2-87 | L25595 | 1a | swArkell | AY115488 | | 3d |
| Hetian88 | D11092 | 1a | T1-China | AJ272108 | | 4a |
| Uchida | D11093 | 1a | HE-JK4 | AB099347 | | 4b |
| Hetian87 | L08816 | 1a | JSN-Sap | AB091395 | | 4b |
| Morocco | AY230202 | 1b | HE-JA1 | AB097812 | | 4b |
| T3-Chad | AY204877 | 1b | swJ13-1 | AB097811 | | 4b |
| Fulm | X98292 | 1a | HE-JI4 | AB080575 | | 4b |
| Mexican | M74506 | 2 | JAK-Sai | AB074915 | | 4c |
| US1 | AF060668 | 3a | JKK-Sap | AB074917 | | 4d |
| US2 | AF060669 | 3a |  |  | |  |
