# Supplementary material for: Calculation of Evolutionary Correlation between Individual Genes and Full-Length Genome: A Method Useful for Choosing Phylogenetic Markers for Molecular Epidemiology
Source: PLoS One. 2013 Dec 3;8(12):e81106. doi: 10.1371/journal.pone.0081106 (PMC3849185; doi:10.1371/journal.pone.0081106)
Supplement: Table S2 — Information about the PCV2 isolates used in this study. (DOC) [file pone.0081106.s005.doc]

**Table S2. Information about the PCV2 isolates used in this study.**

| Accession number | Genotype | Countries of origin | Number in the study |
| --- | --- | --- | --- |
| AB072302.1 | 2A | Japan | 1 |
| AF109398.1 | 2A | Canada | 2 |
| AF117753.1 | 2A | Canada | 3 |
| AY180397.1 | 2B | Taiwan | 4 |
| AY180396.1 | 2B | Taiwan | 5 |
| AY146993.1 | 2B | Taiwan | 6 |
| AY146991.1 | 2B | Taiwan | 7 |
| AF364094.1 | 2B | Taiwan | 8 |
| AY256459.1 | 2C | Hungary | 9 |
| AY256455.1 | 2C | Hungary | 10 |
| AF201310.1 | 2C | Spain | 11 |
| AF201309.1 | 2C | Spain | 12 |
| AF201308.1 | 2C | Spain | 13 |
| AY322004.1 | 2D | France | 14 |
| NC_005148.1 | 2D | Austria | 15 |
| AY424403.1 | 2D | Austria | 16 |
| AY256458.1 | 2D | Hungary | 17 |
| AY256456.1 | 2D | Hungary | 18 |
| AF381176.1 | 2D | China | 19 |
| AF109399.1 | 2D | Canada | 20 |
| AF264043.1 | 2D | USA | 21 |
| AF201306.1 | 2D | Germany | 22 |
| DQ104423.1 | 2E | China | 23 |
| DQ104421.1 | 2E | China | 24 |
| AY325495.1 | 2E | South Africa | 25 |
| AF544024.1 | 2E | Korea | 26 |
| AB072303.1 | 2E | Japan | 27 |
| AF408635.1 | 2E | Canada | 28 |
| AF264042.1 | 2E | USA | 29 |
| AF264040.1 | 2E | USA | 30 |
| DQ141322.1 | 1A | Shandong, China | 31 |
| AY484416.1 | 1A | Netherlands | 32 |
| AY322002.1 | 1A | France | 33 |
| AY424405.1 | 1A | Austria | 34 |
| AY256460.1 | 1A | Hungary | 35 |
| AF201897.1 | 1A | Netherlands | 36 |
| AF201311.1 | 1A | France | 37 |
| AY484407.1 | 1B | Netherlands | 38 |
| AY556475.1 | 1B | Guangxi, China | 39 |
| AY713470.1 | 1C | Germany | 40 |
| AY682996.1 | 1C | Shantou, China | 41 |
| AY035820.1 | 1C | China | 42 |
| AY181947.1 | 1C | Shandong, China | 43 |
