# Supplementary material for: Calculation of Evolutionary Correlation between Individual Genes and Full-Length Genome: A Method Useful for Choosing Phylogenetic Markers for Molecular Epidemiology
Source: PLoS One. 2013 Dec 3;8(12):e81106. doi: 10.1371/journal.pone.0081106 (PMC3849185; doi:10.1371/journal.pone.0081106)
Supplement: Table S3 — Sizes of the individual genes or genomic regions used in this study. (DOC) [file pone.0081106.s006.doc]

**Table S3. The sizes of the individual genes or genomic regions used in study.**

| Genes of JEV | cap | E | NS1 | NS2a | NS2b | NS3 | NS4a | NS4b | NS5 | PreM |
| --- | --- | --- | --- | --- | --- | --- | --- | --- | --- | --- |
| Size (bp) | 381 | 1500 | 1245 | 681 | 393 | 1857 | 378 | 766 | 2715 | 501 |
| Genes of MV | F | H | L | M | N | P | V | C |  |  |
| Size (bp) | 1653 | 1854 | 6552 | 1008 | 1578 | 1524 | 900 | 561 |  |  |
| Genes of HEV | MJ-C | GO | KLY-B | MXJ | SGG-A |  |  |  |  |  |
| Size (bp) | 307 | 186 | 280 | 304 | 371 |  |  |  |  |  |
| Genes for PCV2 | cap | rep |  |  |  |  |  |  |  |  |
| Size (bp) | 702 | 945 |  |  |  |  |  |  |  |  |
