# Supplementary material for: Calculation of Evolutionary Correlation between Individual Genes and Full-Length Genome: A Method Useful for Choosing Phylogenetic Markers for Molecular Epidemiology
Source: PLoS One. 2013 Dec 3;8(12):e81106. doi: 10.1371/journal.pone.0081106 (PMC3849185; doi:10.1371/journal.pone.0081106)
Supplement: Table S4 — Evolutionary correlation r values between the genome and individual genes of MV based on differently sized samples. (DOC) [file pone.0081106.s007.doc]

**Table S4.** Evolutionary correlation r values between the genome and individual genes of MV based on differently sized samples.

| Sample Size | F gene | H gene | L gene | M gene | N gene | P gene | V gene | C gene |
| --- | --- | --- | --- | --- | --- | --- | --- | --- |
| 5 | 0.887 | 0.968 | 0.970 | 0.797 | 0.953 | 0.867 | 0.892 | 0.952 |
| 10 | 0.971 | 0.978 | 0.990 | 0.930 | 0.987 | 0.948 | 0.940 | 0.925 |
| 15 | 0.970 | 0.973 | 0.991 | 0.933 | 0.983 | 0.949 | 0.937 | 0.900 |
| 20 | 0.969 | 0.977 | 0.991 | 0.950 | 0.979 | 0.949 | 0.926 | 0.875 |
| 28 | 0.973 | 0.979 | 0.992 | 0.957 | 0.981 | 0.953 | 0.929 | 0.877 |
