# Supplementary material for: Calculation of Evolutionary Correlation between Individual Genes and Full-Length Genome: A Method Useful for Choosing Phylogenetic Markers for Molecular Epidemiology
Source: PLoS One. 2013 Dec 3;8(12):e81106. doi: 10.1371/journal.pone.0081106 (PMC3849185; doi:10.1371/journal.pone.0081106)
Supplement: Table S5 — Evolutionary correlation r values between the genome and individual genes of PCV2 based on differently sized samples. (DOC) [file pone.0081106.s008.doc]

**Table S5.** Evolutionary correlation r values between the genome and individual genes of PCV2 based on differently sized samples.

| sample size | rep gene | cap gene |
| --- | --- | --- |
| 5 | 0.823 | 0.937 |
| 10 | 0.886 | 0.910 |
| 15 | 0.838 | 0.884 |
| 20 | 0.844 | 0.905 |
| 25 | 0.859 | 0.911 |
| 30 | 0.871 | 0.917 |
| 35 | 0.822 | 0.941 |
| 43 | 0.766 | 0.940 |
