# Supplementary material for: Calculation of Evolutionary Correlation between Individual Genes and Full-Length Genome: A Method Useful for Choosing Phylogenetic Markers for Molecular Epidemiology
Source: PLoS One. 2013 Dec 3;8(12):e81106. doi: 10.1371/journal.pone.0081106 (PMC3849185; doi:10.1371/journal.pone.0081106)
Supplement: Table S6 — Evolutionary correlation r values between the genome and individual genes of JEV based on differently sized samples. (DOC) [file pone.0081106.s009.doc]

**Table S6.** Evolutionary correlation r values between the genome and individual genes of JEV based on differently sized samples.

| Sample size | cap | E | NS1 | Ns2a | Ns2b | NS3 | Ns4a | Ns4b | Ns5 | PreM |
| --- | --- | --- | --- | --- | --- | --- | --- | --- | --- | --- |
| 5 | 0.893 | 0.992 | 0.994 | 0.991 | 0.995 | 0.983 | 0.945 | 0.987 | 0.998 | 0.994 |
| 10 | 0.954 | 0.997 | 0.993 | 0.991 | 0.988 | 0.978 | 0.965 | 0.993 | 0.996 | 0.991 |
| 15 | 0.961 | 0.998 | 0.991 | 0.992 | 0.991 | 0.985 | 0.975 | 0.991 | 0.998 | 0.994 |
| 20 | 0.950 | 0.996 | 0.992 | 0.988 | 0.986 | 0.983 | 0.974 | 0.989 | 0.996 | 0.991 |
| 25 | 0.926 | 0.995 | 0.993 | 0.987 | 0.986 | 0.983 | 0.976 | 0.990 | 0.996 | 0.986 |
| 30 | 0.927 | 0.996 | 0.993 | 0.987 | 0.987 | 0.986 | 0.979 | 0.992 | 0.996 | 0.987 |
