# Supplementary material for: Calculation of Evolutionary Correlation between Individual Genes and Full-Length Genome: A Method Useful for Choosing Phylogenetic Markers for Molecular Epidemiology
Source: PLoS One. 2013 Dec 3;8(12):e81106. doi: 10.1371/journal.pone.0081106 (PMC3849185; doi:10.1371/journal.pone.0081106)
Supplement: Table S7 — Evolutionary correlation r values between the genome and individual genes of HEV based on differently sized samples. (DOC) [file pone.0081106.s010.doc]

**Table S7.** Evolutionary correlation r values between the genome and individual genes of HEV based on differently sized samples.

| Sample size | MJ-C | GO | KLY-B | MJX | SGG-A |
| --- | --- | --- | --- | --- | --- |
| 5 | 0.418 | 0.484 | 0.674 | 0.733 | 0.161 |
| 10 | 0.892 | 0.606 | 0.640 | 0.936 | 0.926 |
| 15 | 0.981 | 0.846 | 0.965 | 0.971 | 0.982 |
| 20 | 0.989 | 0.930 | 0.985 | 0.987 | 0.989 |
| 25 | 0.988 | 0.939 | 0.978 | 0.985 | 0.985 |
| 30 | 0.983 | 0.938 | 0.933 | 0.983 | 0.972 |
