# Supplementary material for: Calculation of Evolutionary Correlation between Individual Genes and Full-Length Genome: A Method Useful for Choosing Phylogenetic Markers for Molecular Epidemiology
Source: PLoS One. 2013 Dec 3;8(12):e81106. doi: 10.1371/journal.pone.0081106 (PMC3849185; doi:10.1371/journal.pone.0081106)
Supplement: Text S1 — Accession numbers for the PCV2 strains used for the calculations based on large numbers of sequences. (DOC) [file pone.0081106.s011.doc]

**Text S1.** Accession numbers for the PCV2 strains used for the calculations based on large numbers of sequences.

The Accession numbers for PCV2 strains with the number of 123:

AF381177.1, FJ644927.1, AY181945.1, FJ644560.1, AF201311.1, HQ378158.1, AY556475.1, AF544024.1, DQ104420.1, FJ804417.1, FJ667583.1, FJ644929.1, AY181948.1, AY321984.1, AY321982.1, HQ148879.1, HM038018.1, HM776445.1, HQ650833.1, AF109398.1, AY321999.1, HM038033.1, KC800641.1, FJ644924.1, AF109399.1, AY682993.1, AY424405.1, AY180397.1, JX534236.1, FJ905469.1, AB072301.1, AY321988.1, AY484414.1, AF264043.1, HM038029.1, AF201309.1, KC533811.1, AY556477.1, AY177626.1, KC800640.1, HM776450.1, KC800637.1, KC688419.1, KC907703.1, AY484407.1, HQ231328.1, FJ905471.1, AY321998.1, GU370064.1, AY180396.1, AY613854.1, JF928004.1, FJ667592.1, AY146991.1, AY322002.1, FJ870971.1, AY256460.1, HM776438.1, KC800646.1, AY256455.1, HM776452.1, AB072303.1, KC533812.1, AY686765.1, AF381176.1, AY510375.1, AY424401.1, AY732494.1, AY686764.1, GU370063.1, AY424403.1, FJ644920.1, AF264040.1, KC153106.1, FJ644562.1, AF381175.1, AY484416.1, HM776453.1, KC800634.1, KC800636.1, KC800638.1, GU247991.1, DQ141322.1, AY682996.1, AF201306.1, AY536756.1, HM776443.1, KC261601.1, AF201308.1, AY256459.1, AY556476.1, HQ231329.1, AY424404.1, HM776442.1, FJ905463.1, FJ594471.1, AY322004.1, GU247992.1, AY682995.1, AF201897.1, AY596823.1, HM038021.1, DQ104421.1, JF827599.1, FJ716703.1, HM038032.1, DQ104423.1, AY325495.1, DQ017036.1, AY288134.1, AY713470.1, GU049342.1, FJ667585.1, FJ644931.1, AY146993.1, AB072302.1, FJ644555.1, AY181946.1, NC_005148.1, FJ905459.1, AF264042.1, AY291317.1, AY484410.1.

The Accession numbers for PCV2 strains with the strains number of 224:

FJ905466.1, FJ644560.1, HQ378158.1, AF201311.1, DQ104420.1, FJ804417.1, FJ667583.1, FJ667594.1, FJ905464.1, AY181948.1, AY321982.1, AY682990.1, FJ667591.1, HM038034.1, HM776445.1, JQ692110.1, FJ644919.1, JQ955679.1, AY321999.1, AY849938.1, FJ644924.1, AY321992.1, KC800641.1, AY682993.1, AY424405.1, AY321986.1, FJ644932.1, AY484414.1, HM038031.1, AY177626.1, KC533811.1, HM776450.1, JF928005.1, KC800637.1, KC688419.1, HQ231328.1, AY321998.1, HM038017.1, AY613854.1, AY321997.1, HM038027.1, JF928004.1, AY146991.1, FJ667596.1, GU247989.1, GU233804.1, AY691679.1, AY686762.1, FJ644922.1, FJ870971.1, HM038020.1, HM776439.1, AY536755.1, AY322001.1, AY256455.1, HM776452.1, KC533812.1, AB072303.1, AY510375.1, AY391729.1, AY732494.1, AY686764.1, FJ905465.1, AY424403.1, HM641752.1, DQ104422.1, AY641542.1, FJ644562.1, AY651850.1, HM038024.1, AF381175.1, HM776453.1, AY484416.1, AY322000.1, FJ644923.1, FJ905460.1, FJ644925.1, HM038016.1, KC800638.1, DQ141322.1, HM776441.1, AY969004.1, AY536756.1, HM776443.1, AY556476.1, AY256459.1, HQ231329.1, AY321987.1, FJ644559.1, HF542107.1, FJ594471.1, AY682995.1, AY291316.1, KC688418.1, AY322003.1, KC800642.1, FJ870975.1, DQ017036.1, AY325495.1, FJ667585.1, AY146993.1, FJ644555.1, AY321995.1, AY181946.1, FJ905459.1, KC800645.1, AY484410.1, FJ667588.1, FJ905467.1, AF381177.1, FJ644927.1, AY181945.1, HM038028.1, AY556475.1, FJ667593.1, HM038030.1, AY682992.1, FJ598045.1, FJ644929.1, AY321984.1, FJ905468.1, HQ148879.1, HM038018.1, EU747085.1, GU049340.1, AY035820.1, AY682997.1, HQ650833.1, AY604430.1, AY484409.1, HM038033.1, AF109399.1, AY180397.1, JX534236.1, HM038023.1, FJ905469.1, AY321988.1, AB072301.1, HM038029.1, FJ644928.1, AY556477.1, AY291318.1, KC800640.1, KC907703.1, FJ667595.1, FJ905462.1, AY484407.1, AY484415.1, FJ905471.1, AY122275.1, AY180396.1, GU370064.1, FJ870973.1, FJ716704.1, FJ667592.1, AY321994.1, AY322002.1, FJ644921.1, AY256460.1, HM776438.1, FJ440338.1, AY484411.1, KC800646.1, AY579893.1, HM776448.1, AY686765.1, AY424401.1, JF928003.1, GU370063.1, GU247990.1, HM776440.1, JF928006.1, AY556474.1, FJ644920.1, KC153106.1, EU408780.1, KC800643.1, HM038019.1, DQ151643.1, KC800634.1, HQ378159.1, KC800636.1, AF408635.1, AY682996.1, GU247991.1, AY094619.1, HQ378160.1, AY678532.1, KC261601.1, FJ644563.1, AY424404.1, HM776442.1, GU247988.1, FJ905463.1, AY322004.1, GU247992.1, HM776449.1, FJ644557.1, HM038021.1, AY596823.1, AY256458.1, FJ870970.1, JF827599.1, AY424402.1, KC261600.1, FJ716703.1, HM038032.1, AY484412.1, AY321990.1, FJ644556.1, FJ644930.1, AY288134.1, AY484413.1, KC800644.1, GU049342.1, FJ644931.1, AY321996.1, AY682994.1, AB072302.1, HM038022.1, FJ644926.1, AY321985.1, FJ905470.1, AY291317.1.
